# Development and Testing of a Long-Term Care Cost Planning and Education Tool for Caregivers

**DOI:** 10.1093/geroni/igaf122.011

**Published:** 2025-12-31

**Authors:** Louise (Siyu) Gao, Kelly Moeller, Marguerite DeLiema

**Affiliations:** University of Minnesota, Minneapolis, Minnesota, United States; University of Minnesota Twin Cities, Minneapolis, Minnesota, United States; University of Minnesota School of Social Work, Minneapolis, Minnesota, United States

## Abstract

An estimated 52% of Americans over 65 years old will need high levels of long-term care late in life (Favreault & Dey, 2016), but most have not planned for their long-term care needs nor saved enough to cover the high cost of long-term care (AP-NORC Center for Public Affairs Research, 2014). Medicaid provides a critical safety net for those who have exhausted their savings and require skilled care, but the complexity of Medicaid eligibility rules and covered care options create barriers to enrollment. We developed “Care Assistant,” the first website to provide state-specific and personalized Medicaid enrollment timelines for older adults who need long-term services and supports (LTSS). Designed to reduce information overload for informal caregivers, the website alleviates caregiver burden by providing information on what financial resources the care recipient may qualify for. In this study, 112 caregivers of adults aged 60+ interacted with and completed tasks on Care Assistant (mean age = 43). Participants completed a Medicaid knowledge test before and after the website testing session, which was video-recorded and analyzed using rapid qualitative insights and descriptive statistics. Results indicate that using the website and watching four Medicaid explainer videos significantly improved participants’ Medicaid program knowledge (t = 13.3, p<.001). Participants found it easy to enter the care recipient’s financial information to determine their Medicaid eligibility (mean= 5.9/7) and the Care Cost Calculator was particularly helpful in learning about their local LTSS costs. Results indicate that a personalized, easy-to-navigate website can ease the burden of care planning.

